# Low Solar Absorptance, High Emittance Performance Thermochromic VO_2_-Based Smart Radiator Device

**DOI:** 10.3390/nano12244422

**Published:** 2022-12-11

**Authors:** Ali Hendaoui

**Affiliations:** Physics Department, College of Science and General Studies, Alfaisal University, P.O. Box 50927, Riyadh 11533, Saudi Arabia; ahendaoui@alfaisal.edu

**Keywords:** thermochromic VO_2_, tunable emittance, cold mirror, solar absorptance, smart radiator device, energy efficiency, thermal control coatings for spacecrafts, nanosatellites

## Abstract

Thermochromic vanadium dioxide (VO_2_)-based smart radiator devices (SRDs) display emittance variation with changes in temperature, making them very promising for energy-efficient thermal control of spacecrafts in general, and nanosatellites in particular. However, the high solar absorptance of the VO_2_-based SRDs remains too high for their intended application. Based on an approach combining optical simulation and experimental work, I demonstrate that an additional top stack layer alternating between high and low refractive indices made of a-Si(25 nm)/SiO_2_(67 nm) reduces the solar absorptance of a VO_2_-based SRD by 35% (from 0.43 to 0.28) while keeping the emittance performance of the SRD within the requirements for the intended application (low-temperature emittance $\varepsilon_{L}$ = 0.35, high-temperature emittance $\varepsilon_{H}$ = 0.81 and emittance tuneability with temperature Δε = 0.46). I also discuss factors to consider while designing additional top stack layers alternating between high and low refractive indices to further decrease the SRD’s solar absorptance without affecting its emittance performance.

## 1. Introduction

Smart radiator devices (SRD) are a very promising alternative to the mechanical louver for energy-efficient thermal management of spacecrafts in general, and nanosatellites in particular [1]. The operation of an SRD is based on its adaptive emittance, which changes with temperature (these changes can later be reversed), allowing it to reject more heat at high temperatures and less heat at low temperatures to help keep the interior of the satellite within a temperature range suitable for its operation (usually around room temperature) [2].

Requirements for an effective SRD for the thermal management of spacecrafts include (i) the highest possible emittance at high temperatures ($\varepsilon_{H}$ > 0.8), (ii) the largest possible tuneability at the temperature (Δε > 0.4) and (iii) the lowest possible solar absorptance (α < 0.2) [3]. These requirements are based on the fact that the temperature of a satellite is the result of a balance between heat generated by its internal systems, heat input due to solar absorptance and heat rejection due to emittance. Lowering the solar absorptance would reduce the heat input and therefore increase the cooling effect via heat radiation through its emittance.

Thermochromic vanadium dioxide, VO_2_, is a particularly interesting material because of the significant changes occurring in its electrical and optical properties (i.e., high tuneability) during the insulator-to-metal transition, which happens at approximately 68 °C [4]. This transition, which occurs on the scale of one hundred femtoseconds [5], is accompanied by a structural change from the low-temperature monoclinic phase to the high-temperature tetragonal phase. However, for some applications, it is necessary to have a transition temperature close to room temperature. Research has shown that it is possible to change the transition temperature by incorporating doping elements into the material. High-valence cations, for example Nb^5+^, Mo^6+^ and W^6+^ [2,6], act as donors (n-type dopants), and when VO_2_ is doped with these elements, the transition temperature decreases. VO_2_ is therefore an excellent material for many applications, particularly in ultra-fast switching [7] and smart windows [8].

Since the potential applications of VO_2_ involve their use as thin films, several techniques have been used for the deposition of thin films of VO_2_. Among them, pulsed laser deposition (PLD) remains one of the most suitable techniques as it ensures the synthesis of VO_2_ films with good thermochromic properties in a reproducible way without a post-annealing step [1,2,9]. Other techniques, such as sputtering [6] and chemical vapor deposition [10], are also reported for preparing VO_2_ films, while in most cases, a deposition temperature (i.e., of the substrate) above 400°C is required to obtain a high contrast in the optical and electrical properties of VO_2_ thin films on either side of the transition. A recent report by Vlcek et al. on reactive high-power impulse magnetron sputtering (HiPIMS) demonstrated the possibility of preparing VO_2_ films with excellent thermochromic properties and a low thermochromic transition temperature at low substrate temperatures (300 °C) on amorphous soda-lime glass substrates [11]. Another report from the same group showed that it is possible to deposit crystalline VO_2_ films on crystalline Si at 250 °C [12]. More recently, Kolenaty et al. [13] demonstrated that when an HiPIMS-deposited VO_2_ film was doped with W, multilayer smart windows with a thermochromic transition temperature of around 20–21 °C could be fabricated. Doping with W involved the use of DC sputtering concurrently with HiPIMS, offering more flexibility in terms of controlling the doping content in VO_2_ films, and the processing temperature of the W-doped VO_2_ was 330 °C. These results are important not only for the preparation of smooth films needed in optical devices, as demonstrated by Kolenaty et al., but also because magnetron sputtering is a well-established method in the industry for the preparation of films over large-surface substrates, including temperature-sensitive ones (i.e., scaling up at a low cost) [8]. Several reports can be found in the literature regarding the control of the emittance of VO_2_-based structures for applications such as smart radiator devices. Benkahoul et al. demonstrated a sputter-deposited 300 nm thick VO_2_ film on an Al substrate that exhibited a change in the emittance from 0.2 to 0.42 across the transition temperature [14]. Such emittance behavior, referred to as “positive emittance switching”, is suitable for smart radiator device applications, in contrast with the behavior observed for VO_2_ films deposited on IR-transparent substrates (such as silicon), where the emittance switches from a high value at low temperature to a high value at high temperature. Another report by Voti et al. describes different simulated VO_2_-based structures with an emittance that experiences dramatic changes as a result of reaching temperatures in the so-called mid-wave IR (MWIR) range for military applications (i.e., furtivity) [15].

Since their development by our group [1,2,16], thermochromic VO_2_-based SRDs, consisting of a smart Fabry–Perot resonant cavity comprising a bottom IR-reflective layer (gold), an IR-transparent dielectric optical gap (SiO_2_) and a very thin thermochromic VO_2_ layer on top, have sparked interest as they display an unprecedented combination of a high emittance at high temperatures, large tuneability and integrability on the existing parts of nanosatellites. Furthermore, VO_2_-based SRDs are very light, with a mass of a few grams / m^2^, making them suitable for nanosatellites. Wang et al. improved the emittance tuneability (up to Δε = 0.55) by using HfO_2_ as the optical gap layer as it displays high transparency in the mid-infrared range (until 12 µm) compared to SiO_2_ [17]. However, such an improvement in Δε was accompanied by a decrease in $\varepsilon_{H}$ to 0.7, which might be detrimental to the heat rejection capability of SRDs at high temperatures. Capitalizing on a plasmonic effect generated at high temperatures, Sun et al. demonstrated the possibility of improving the emittance tuneability Δε of an SRD comprising a continuous 50 nm thick VO_2_ film from 0.37 to 0.48 using a patterned VO_2_ film-based meta-reflector [18]. The authors also reported a decrease in the solar absorptance of the device as a result of the partial coverage of patterned VO_2_ films. However, the solar absorptance at 25 °C reported in the study was still above 0.4 for the meta-reflectors, displaying the Δε needed for SRD applications (>0.4). Overall, the scientific literature shows that work on VO_2_-based SRDs remains mainly focused on the improvement of the infrared emittance performance via the appropriate choice of gap material or thickness or patterning VO_2_ films [2,19,20]. However, while the relatively high solar absorptance of the VO_2_-based coating remains a major limiting factor for its technological application, studies related to its reduction are scare. Therefore, studies are needed to elaborate a clear and technologically viable solution for this lingering matter. 

Cold mirrors are a type of distributed Bragg reflector (DBR) that are highly reflective in the visible light band and highly transmissive in the infrared range [21]. Such mirrors are designed using alternating layers of high–low refractive indices. Each layer has an optical thickness of about a quarter of the center wavelength for maximum reflection (λ/4). A sequence of two consecutive high–low refractive indices λ/4 layers is called a stack layer or pattern. This approach is promising not only because it allows a high reflectance (i.e., DBR stopband) throughout the visible range by controlling the optical thicknesses and the number of alternating layers (i.e., patterns) to target the reflection of different wavelengths, but also presents the opportunity to achieve a large stopband width if the contrast between the refractive indices is important [22]. The latter is expected to help in reducing the number of layers in cold mirrors to achieve a reflection over larger bands of wavelengths. A lower number of layers in a cold mirror is expected to reduce the complexity of the structure, lower the fabrication costs and increase their integrability with existing devices. 

In this work, based on the principle of cold mirrors, a promising approach for effectively reducing the solar absorptance of VO_2_-based SRDs while maintaining the necessary emittance performance in the infrared range is proposed. Combining optical simulation and experimental work, I demonstrate that an additional top stack layer (i.e., pattern) made of a-Si/SiO_2_ λ/4 layers helps reduce the solar absorptance of the VO_2_-based SRD structure by 35% (from 0.43 to 0.28) while keeping its emittance performance within the requirements for the intended application ($\varepsilon_{H}$ = 0.81 and Δε = 0.46). An extensive discussion on the factors that should be taken into consideration for properly integrating “cold mirrors” in VO_2_-based SRDs to achieve an effective reduction in solar absorptance is provided. These factors include (i) the proper choice of materials for the fabrication of multilayer cold mirrors in terms of their refractive indices and compatibility for space applications, (ii) the optimization of the thickness of the layers to ensure an effective decrease in the solar absorptance around the maximum of the solar spectrum and (iii) the appropriate device fabrication process. 

## 2. Materials and Methods

### 2.1. Optical Design

Optical simulation studies on the solar reflectance were carried out using OpenFilters software [23]. Au, SiO_2_ and Si layers were described by well-defined optical constants from the software database. The optical constants used to describe the VO_2_ layer were extracted from ref. [24].

Several factors must be considered in designing an effective distributed Bragg reflector [25]. First, it is important to alternate high / low refractive indices layers to alternate the sign of the amplitude reflection coefficient between two consecutive reflections. The amplitude reflection coefficient, r, for an electromagnetic wave propagating from a medium 1 to a medium 2 at a normal incidence is given by:

$$r=\frac{n_{2}-n_{1}}{n_{2}+n_{1}}$$

where $n_{1}$ and $n_{2}$ are the refractive indices of medium 1 and medium 2, respectively. It is important to mention that a sequence medium 1/medium 2 is called a pattern or a stack. If $n_{2}\;$> $n_{1}$, the amplitude reflection coefficient is positive and no change in the phase takes place when the incident beam is reflected at the interface (i.e., the reflected wave has the same phase as the incident one). However, if $n_{2}$ < $n_{1}$, the amplitude reflection coefficient is negative, and the reflected wave experiences a π phase shift with respect to the incident wave. Second, the optical path between two consecutive reflections should be half of the targeted wavelength. This means that the optical thickness of each layer should be a quarter of the targeted wavelength. Through a combination of alternating signs of the amplitude reflection coefficient at the interfaces and an optical thickness of a quarter target wavelength for each layer, all the beams reflected at the different interfaces will emerge in-phase and interfere constructively, resulting in a high reflection. If more patterns are added to the mirror, the reflection becomes higher. Finally, the contrast between the refractive indices of two consecutive layers should be as large as possible to increase the reflectance at the interfaces between the layers and the reflection bandwidth.

### 2.2. Deposition of the Films and Fabrication of the Optimized SRD Structure

Conventional magnetron sputtering was used to deposit the reflective gold layer (i.e., mirror) on a quartz substrate at room temperature (20 to 25 °C). Plasma-enhanced chemical vapor deposition (PECVD) was used to deposit both silicon dioxide SiO_2_ and amorphous silicon, a-Si, using well-established procedures at relatively low temperatures (390 °C and 350 °C for SiO_2_ and a-Si, respectively). It is worth mentioning that low-temperature processing of films in a multilayer structure is suitable to avoid temperature-induced modification of the films (change in the composition, crystallinity, etc.) as well as to minimize any interdiffusion between the layers. The most challenging part of the SRD fabrication was the deposition of a very thin VO_2_ thermochromic layer with good thermochromic properties in the mid-IR range, which is necessary to ensure optimal emittance modulation properties with the temperature. This was achieved using reactive pulsed laser deposition (RPLD) at a substrate temperature of 450 °C. The main reason why RPLD was used to deposit VO_2_ was to ensure the reproducibility of high-quality VO_2_ films. The details of the processing parameters of VO_2_ films are given in reference [26]. Low deposition rates are needed to ensure the deposition of continuous layers, especially for low thicknesses. While the thickness of the Au layer was controlled using a quartz microbalance integrated into the sputtering systems, the thicknesses of a-Si, SiO_2_ and VO_2_ were controlled via the deposition rate. More specifically, thick amorphous films were first deposited on sacrificial substrates for a given deposition time. After that, the thicknesses of the films were measured using cross-section scanning electron microscopy images. The deposition rates of SiO_2_, VO_2_ and a-Si films, calculated as thickness/deposition time, were used to control their thicknesses by controlling the deposition time of each layer during the fabrication of the SRD multilayer structure. More details about the determination of the deposition rates of a-Si, SiO_2_ and VO_2_ films can be found in the Supplementary Materials.

### 2.3. Characterization

X-ray diffraction, XRD (PANalytical’s X’Pert PRO MRD diffractometer, Cu Kα radiation), was used to monitor the crystallinity and the phase purity of the VO_2_ film since these characteristics are directly correlated with its thermochromic properties. Atomic force microscopy, AFM (Bruker Dimension Icon, Berlin, Germany), was used to characterize the surface morphology of the SRD before and after the deposition of the stack layer. The roughness of the films is reported as a root-mean-square (RMS) value since it is more sensitive to the peaks and valleys on the depth profile [27]. A JEOL JSM-6300F scanning electron microscope (SEM) was used for the determination of the deposition rates of a-Si, SiO_2_ and VO_2_ films (cf., Section 2.2 and Supplementary Materials).

The emittance was calculated from the total reflectance of the SRD using the equation

$$\varepsilon \left(T\right)=\frac{\int_{\lambda_{1}}^{\lambda_{2}}\left(1-R_{\lambda ,IR}\left(T\right)\right)P\left(\lambda ,T\right)d\lambda }{\int_{\lambda_{1}}^{\lambda_{2}}P\left(\lambda ,T\right)d\lambda }$$

where $R_{\lambda ,IR}\left(T\right)$ is the spectral reflectance measured at near-normal incidence using a Nicolet 6700 FTIR spectrometer at room temperature and at 25 °C and 100 °C in the wavelength range from $\lambda_{1}=$ 2.5 µm to $\lambda_{1}=$ 25 µm, and P(λ,T) stands for the radiation of a blackbody at temperature ***T*** given by Planck’s function for the given wavelength and temperature.

The solar absorptance α was calculated for both simulated and measured spectral reflectance at room temperature using the equation

$$\alpha =\frac{\int_{250\;nm}^{1800\;nm}\left(1-R_{\lambda ,solar}\right)AM0\left(\lambda \right)d\lambda }{\int_{250\;nm}^{1800\;nm}AM0\left(\lambda \right)d\lambda }$$

where $R_{\lambda ,solar}$ is the spectral reflectance and AM0(λ) is the air mass 0 solar irradiance (ASTM E490) received by the satellite in space [28]. The experimental values of $R_{\lambda }$ were obtained using a PerkinElmer Lambda19 spectrophotometer equipped with an integrating sphere to measure the total reflectance. 

## 3. Results and Discussion

### 3.1. Multilayer VO_2_/SiO_2_/Au SRD Experimental Characterization

#### 3.1.1. VO_2_ Layer Phase Purity and Crystallinity

**Figure 1:**
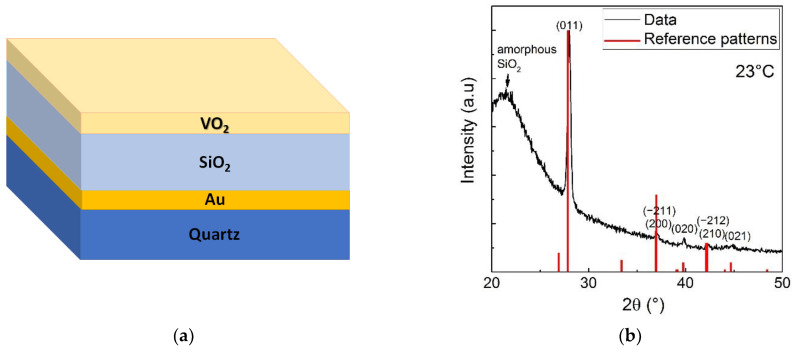
(**a**) Schematic illustration of the multilayer SRD structure (not to scale) consisting of VO_2_(20 nm)/SiO_2_(1330 nm)/Au(300 nm) and (**b**) XRD patterns of a VO_2_ film deposited on SiO_2_ optical gap layer.

VO_2_ phase purity and crystallinity are important parameters in achieving a large variation in infrared reflectance as a function of the temperature (i.e., tuneability). Therefore, it is important to crystallize VO_2_ as a single phase free of any other V_x_O_y_ oxides [29]. After the fabrication of the SRD structure shown in Figure 1a, XRD was performed on it. The corresponding results, shown in Figure 1b, reveal the formation of a pure VO_2_ monoclinic (i.e., low temperature) phase (JCPDS Card No. 44-0252) with a preferential (011) orientation (peak at 2θ ≈ 28.0°). The average size of VO_2_ crystallites was estimated to be about 24 nm using Scherrer’s formula, which indicates a high crystallinity of the film despite its low thickness. The XRD patterns also show a broad feature around 2θ ≈ 21.5° characteristic of amorphous SiO_2_. The results are in accordance with the literature on the growth of VO_2_ films on SiO_2_ [26]. 

#### 3.1.2. Surface Morphology of the SRD Layers

**Figure 2:**
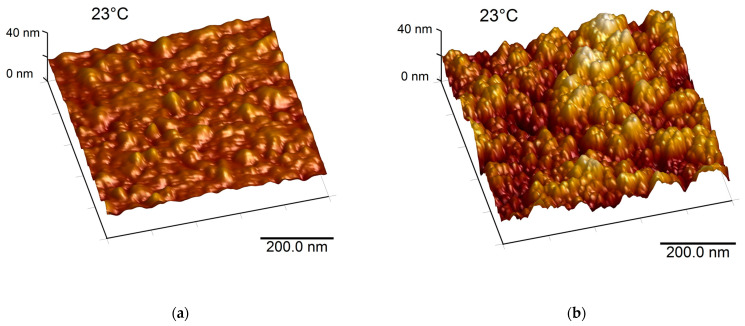

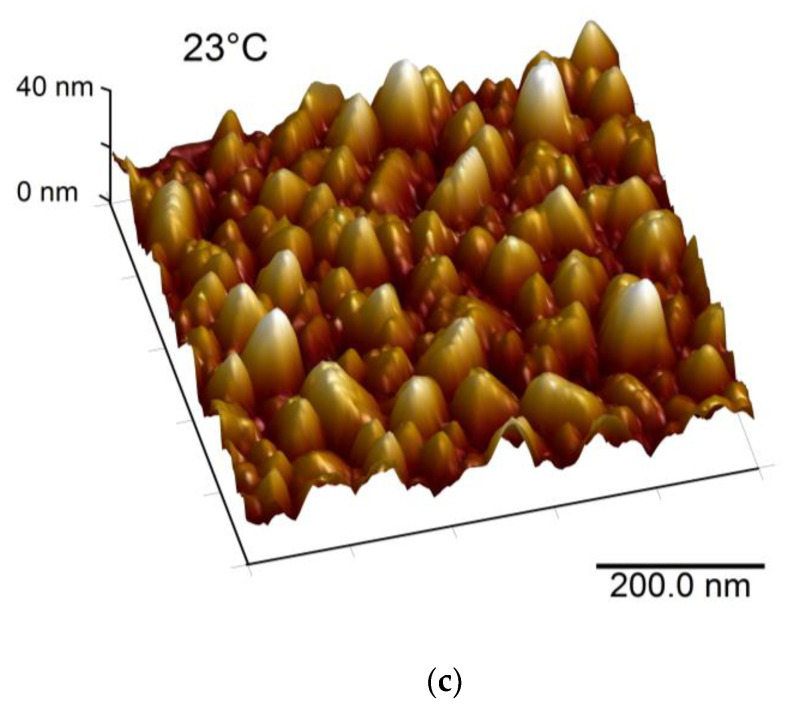
AFM images of the layers: (**a**) Au (300 nm-thick on quartz); (**b**) SiO_2_ (1330 nm-thick on Au); and (**c**) VO_2_ (20 nm-thick on SiO_2_).

When designing optical devices, the roughness of each of the layers included in the device is an important parameter expected to impact the device’s optical properties in the UV–Vis–NIR range. High surface roughness is known to increase the absorptivity [30]. In this sense, the surface morphology of single layers was investigated using AFM. The results are shown in Figure 2.

The Au layer (300 nm-thick) deposited by sputtering at room temperature displayed a relatively smooth surface with an RMS roughness of about 1.45 nm. This value is very close to that of the quartz substrate (around 1nm) since the low-temperature processing of the layer does not allow for the incoming sputtered Au adatoms to diffuse over large lengths on the substrate. As for the 1330 nm-thick SiO_2_ layer grown by PECVD at 390 °C (cf., Figure 2b), the surface morphology displayed islands formed of nanometric clusters, with an RMS roughness of about 4.18 nm. During the PECVD process, high-mobility Si_x_O_y_ clusters are formed and their related diffusion length increases, which in turn increases the roughness [31]. The surface morphology of the 20 nm-thick VO_2_ showed the formation of grains indicating an island-like growth of the films (cf., Figure 2c). The RMS roughness of the VO_2_ layer was 5.39 nm. While a low deposition rate and high substrate temperature are needed to ensure a good crystallinity of the VO_2_ layer, which in turn is needed to have good switching properties of VO_2_, such parameters favor the growth of island-like structure of VO_2_ with a preferred (011) orientation, resulting in high roughness of the VO_2_ film [26]. 

#### 3.1.3. Characterization of the SRD Emittance Performance

**Figure 3:**
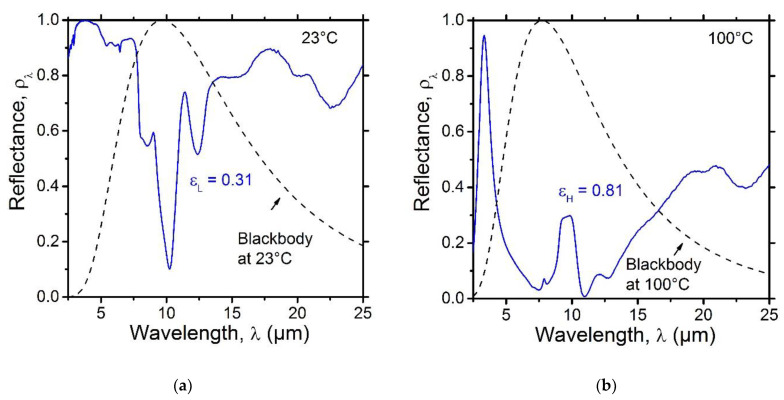
Measured mid-IR spectral reflectance of the VO_2_(20 nm)/SiO_2_(1330 nm)/Au(300 nm) SRD (**a**) at 23 °C and (**b**) 100 °C.

Figure 3 shows the mid-infrared spectral reflectance of the SRD at 23 °C (Figure 3a) and 100 °C (Figure 3b). At 23 °C, the VO_2_ layer was in its semiconducting transparent state and the SRD displayed a high infrared spectral reflectance as it operated mainly as an infrared radiation reflector, except for the absorption bands of SiO_2_. The emittance at a low temperature (i.e., 23 °C) was $\varepsilon_{L}$ = 0.31. At 100 °C, the VO_2_ layer was in its metallic semi-reflective state and the SRD was then operating as a reflection interference filter, resulting in a low reflectance around the peak of the blackbody emission at 100 °C. The emittance at high temperature (i.e., 100 °C) was $\varepsilon_{H}$ = 0.81. The tuneability range of the emittance was $\Delta \varepsilon =\varepsilon_{H}-\varepsilon_{L}$ = 0.50. Such an emittance performance fulfills the requirements for thermal management coating for spacecrafts [3]. 

#### 3.1.4. Characterization of the SRD Solar Absorptance

**Figure 4:**
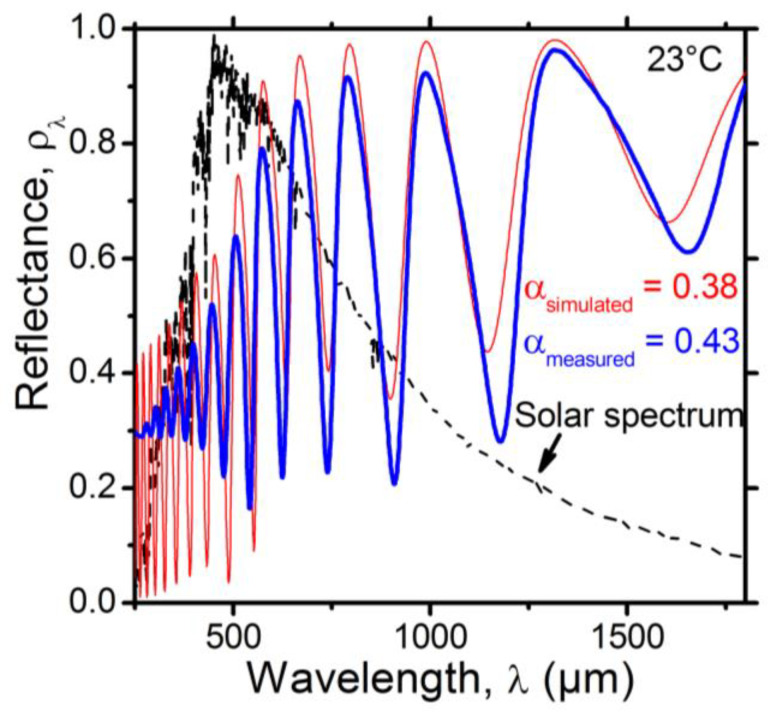
Comparison between the experimental (thick blue line) and the simulated (thin red line) UV–Vis–NIR spectral reflectance of the VO_2_(20 nm)/SiO_2_(1330 nm)/Au(300 nm) SRD. The dotted black line represents the normalized air mass 0 (AM0) solar irradiance (ASTM E490).

The measured UV–Vis–NIR spectral reflectance of the SRD is shown in Figure 4 as a thick blue line. The experimental solar absorptance of the SRD was $\alpha_{measured}=0.43$. This value is above what is acceptable for a thermal management device for space applications. In order to validate the prediction model, the result of the simulation of the UV–Vis–NIR spectral reflectance of the SRD was added and is represented as a thin red line in Figure 4. One can observe that the trend between the experimental results and the simulation is confirmed, with a simulated solar absorptance of $\alpha_{simulated}=0.38$.

It is worth mentioning that some differences between the simulation and experimental results could be noticed, especially at short wavelengths. Such differences could be the result of one or more factors. First, while the simulation assumed the use of continuous and smooth layers, the processing of the films induced roughness, as indicated by the AFM results in Figure 2. The roughness at the different interfaces between the deposited layers is expected to increase the absorptance, especially at short wavelengths. Second, the optical properties of the layers in general, and VO_2_ in particular, could experience some changes due to the processing of additional layers, especially at high temperatures. For example, VO_2_ films’ morphological, electrical and optical properties could be modified by controlling the substrate temperature [26]. Third, interdiffusion between adjacent layers could take place. Fourth, the simulation used optical constants from the literature. The actual optical constants of our layers may have been slightly different, especially in the UV range of wavelengths. 

Capitalizing on the results shown in Figure 4, we decided to adopt simulation as a tool for predicting the evolution trend of the spectral reflectance of the SRD after the addition of a top distributed Bragg reflector. 

#### 3.1.5. Design of the Additional Top Distributed Bragg Reflector

In designing the stack layers to be incorporated into the SRD as a distributed Bragg reflector, I assumed a 0° incidence angle of unpolarized light. The objective was to increase the spectral reflectance in the spectral range of 420 nm to 450 nm, as targeting this range offers a good compromise between attempting to improve spectral reflectance in the spectral region where the lowest values of the spectral reflectance are measured for the SRD and its proximity to the spectral range with high solar irradiance values. Moreover, as the angle of incidence increases, the spectral range over which the reflection takes place is expected to shift to longer wavelengths, bringing it closer to the maximum solar irradiance. 

**Figure 5:**
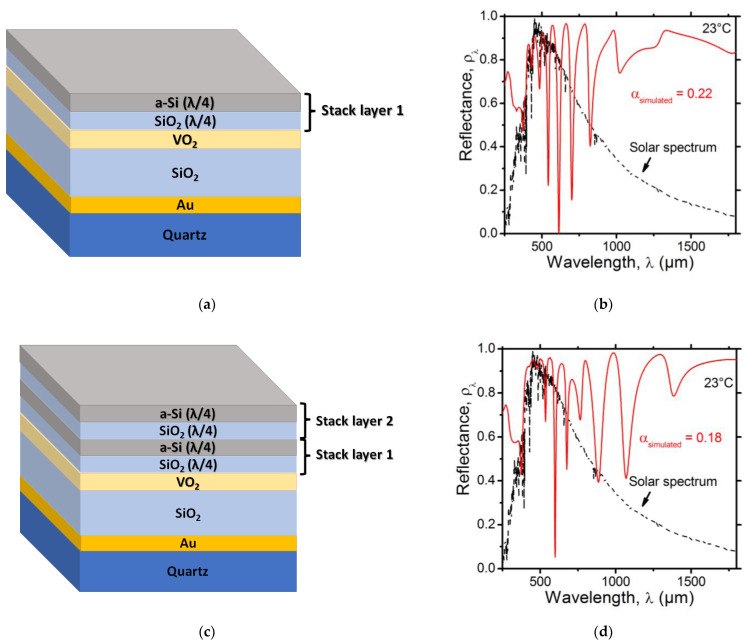
(**a**) Schematic illustration of the SRD with one extra top a-Si(25 nm)/SiO_2_(67 nm) stack layer (not to scale), (**b**) simulation graph of its UV–Vis–NIR spectral reflectance, (**c**) schematic illustration of the SRD with two extra top a-Si(25 nm)/SiO_2_(67 nm) stack layers (not to scale), (**d**) simulation graph of its UV–Vis–NIR spectral reflectance. The dotted black line represents the normalized air mass 0 (AM0) solar irradiance (ASTM E490).

As for the materials used for the individual layers, I decided to consider SiO_2_ and a-Si deposited by PECVD not only because they are both readily available as they are widely used in technological applications, but also due to the important contrast between their refractive indices, allowing the achievement of the desired reflection over a large bandwidth [8]. In addition, SiO_2_ acts as a protective barrier layer between VO_2_ and a-Si [32]. Another feature that could be an additional benefit for selecting a-Si deposited by PECVD (i.e., hydrogenated a-Si) is its good tolerance to electron and proton irradiation effects, which is expected to provide SRDs with additional protection in space [33]. Figure 5a provides an illustration of an SRD with an extra reflective top stack layer made of Si(25 nm)/SiO_2_(67 nm). The simulation of its UV–Vis–NIR spectral reflectance, shown in Figure 5b, indicates an increase in the reflectance. The corresponding solar absorptance is expected to drop ($\alpha_{simulated}=0.22$). Figure 5c provides an illustration of an SRD with a second top stack layer. As can be seen in Figure 5d, the UV–Vis–NIR spectral reflectance was stronger around the target wavelength range, and the calculated solar absorptance further dropped to $\alpha_{simulated}=0.18$.

#### 3.1.6. Surface Morphology and Solar Absorptance of the SRD with a Top Stack Layer

**Figure 6:**
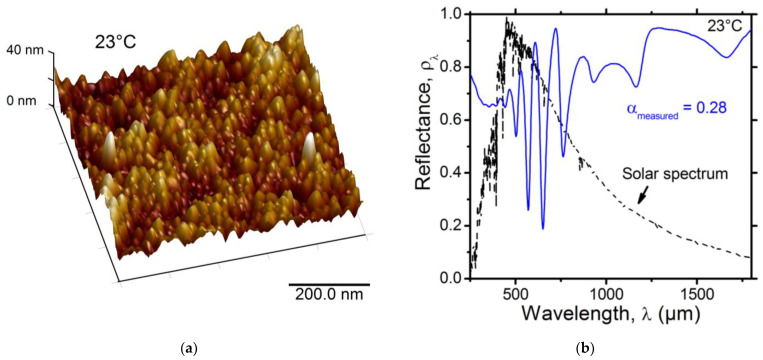
(**a**) AFM image of the surface of the SRD with an extra top a-Si(25 nm)/SiO_2_(67 nm) stack layer and (**b**) experimental graph of its UV–Vis–NIR spectral reflectance. The dotted black line represents the normalized air mass 0 (AM0) solar irradiance (ASTM E490).

The AFM image of the surface of the SRD with a top a-Si/SiO_2_ layer, presented in Figure 6a, shows island-type morphology due to nanometric clusters with an RMS roughness of about 4.52 nm. Such morphology is similar to what was observed for the SiO_2_ optical gap layer in the SRD (cf., Figure 2b) and seems to be mainly dictated by the SiO_2_ layer, since a-Si is very thin and tends to produce low roughness [34].

The UV–Vis spectral reflectance of the SRD with a top a-Si/SiO_2_ layer is given in Figure 6b. As predicted, an increase in the reflectance was observed around the maximum of the AM0 solar irradiance spectrum. The experimental solar absorptance of the device dropped to $\alpha_{measured}=0.28$. This represents a decrease of 35% in the solar absorptance due to the addition of the stack layer. However, it is worth mentioning that a discrepancy between the model and the experimental results was still observed. The possible reasons for such a discrepancy were discussed in Section 3.1.3. 

#### 3.1.7. Characterization of the Emittance Performance of the SRD with a Top Stack Layer

**Figure 7:**
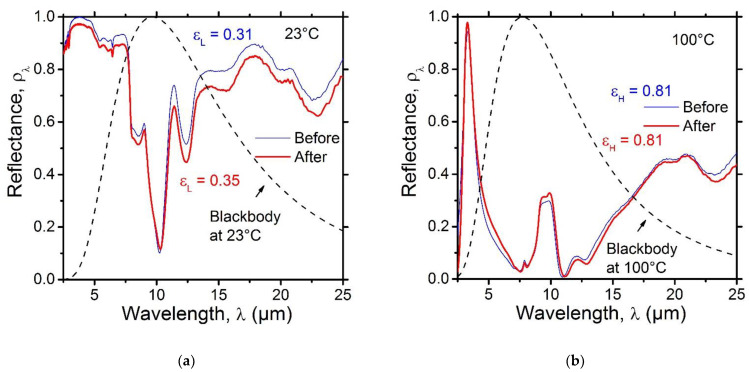
Comparison between the measured mid-IR spectral reflectance of the SRD before (thin blue line) and after (thick red line) adding a λ/4 a-Si(25 nm)/SiO_2_(67 nm) stack layer (**a**) at 23 °C and (**b**) 100 °C.

Figure 7 shows the mid-infrared spectral reflectance of the SRD with a top a-Si/SiO_2_ stack layer at 23 °C (thick red line in Figure 3a) and at 100 °C (thick red line in Figure 3b). The mid-infrared spectral reflectance of the SRD prior to the deposition of the extra stack layer was added for comparison (thin blue line). The results show that the operation of the optimized SRD in the infrared range is still above the requirements for thermal control applications in spacecrafts, with an emittance at low temperature (i.e., 23 °C) of $\varepsilon_{L}$ = 0.35 and an emittance at high temperature (i.e., 100 °C) of $\varepsilon_{L}$ = 0.81. The tuneability range of the emittance was $\Delta \varepsilon =\varepsilon_{H}-\varepsilon_{L}$ = 0.46. While adding an extra a-Si/SiO_2_ stack did not seem to affect the high-temperature emittance of the SRD, because the operation of the device is based on the interference taking place at the surface of VO_2_ layer when it is in its metallic (i.e., IR reflective) state, the low-temperature emittance was slightly increased, mainly due to extra absorption from the absorption bands of SiO_2_. A similar observation was made in a previous study by our group when using a 1570 mm thick SiO_2_ layer as the optical gap [2]. In order to optimize both the solar absorptance and IR emittance of the SRD, it is vital to consider such an increase in the low-temperature emittance of the SRD in its initial design (i.e., prior to the deposition of VO_2_/SiO_2_/Au). Additionally, oxides other than SiO_2_ could be explored as both optical gap and solar reflector layers based on their absorption in the mid-infrared range. It is worth mentioning that, while other IR-transparent materials such as fluorides could be perceived as a better option as compared to oxides due to their high transparency in the mid-infrared range, the lack of stability of fluorides in the space environment and their poor mechanical properties remain major barriers to their effective use in space applications [26,35]. 

Prior to the emergence of VO_2_-based SRDs, studies on the development of SRDs were mainly based on doped perovskite lanthanum manganese oxide (LaMnO_3_, LMO). In 2001, Shimazaki et al. revealed that the insulator-to-metal transition in bulk La_0.825_Sr_0.175_MnO_3_ (dimensions 30 × 30 × 0.2 mm) resulted in an emittance tuneability of Δε=0.42 (from 0.2 to 0.62) in the temperature range −100 °C (i.e., 173K) to 7 °C (i.e., 280K) [36]. Tachicawa et al. demonstrated the stability of the emittance performance of La_0.825_Sr_0.175_MnO_3_ and La_0.7_Ca_0.3_MnO_3_ bulky ceramic tiles (dimensions 30 mm × 30 mm × 200 µm) under proton, electron and UV irradiation [37]. Since doped LMO-based SRDs suffer from high solar absorbance, often exceeding 0.8, Tachikawa et al. [38] used a generic algorithm to design a multilayer structure on top of La_0.775_Sr_0.115_Ca_0.11_MnO_3_ (dimensions 30 mm × 30mm × 70 µm) to decrease its solar absorbance from 0.81 to 0.22. To achieve this, eight layers of a-Si, MgF_2_ and Ge were needed. No details about the thicknesses of the layers could be found in their article. Flight demonstrations of optimized La_0.775_Sr_0.115_Ca_0.11_MnO_3_ (dimensions 30 mm × 30 mm × 70 µm) ceramic tiles on spacecrafts launched by the Japan Aerospace Exploration Agency confirmed a reduction in the SRD’s temperature while saving heater power [39]. This being said, SRD technology based on doped LMO materials still seems to suffer from limitations in its optimal use for spacecrafts in general, and nanosatellites in particular. Doped LMO-based ceramic tiles are bulky and brittle, which limits their integrability on the satellite and increases the risk related to their machine- and/or launch-induced vibration. The solution to this could be using doped LMO material in the form of thin films instead of ceramic tiles. Unfortunately, the emittance performance of doped-LMO films tends to be lower than that of ceramic tiles, and many recent studies have primarily focused on the improvement in the emittance performance of doped LMO-based perovskites when prepared in the form of thin films [39]. Other limitations, such as the wide metal–insulator transition ranges and slow transition speeds, must be tackled for effective use of doped LMO-based SRDs as passive thermal control systems for nanosatellites [40].

In contrast, VO_2_-based SRDs display a combination of characteristics, making them suitable for space applications in general, and for nanosatellites in particular. First and foremost, the VO_2_-based SRD has a total thickness of less than 2 µm, making it lightweight. Moreover, it is perfectly integrable onto the existing parts of satellites as it is based on thin film technology. As for its solar absorbance, it has a value of about 0.43, which is around half that of its doped LSO-based counterpart. As demonstrated in the present work, the solar absorbance could be decreased to 0.28 with only one additional top a-Si/SiO_2_ stack reflector, and the simulation results show that doubling this stack would decrease the solar absorbance to below 0.2 (Figure 5d). Of course, the fabrication process of the SRD must be optimized toward minimizing the roughness of the layers before attempting the addition of the second a-Si/SiO_2_ stack. Another aspect that plays in favor of VO_2_-based SRDs is their narrow transition range, dictated by the sharpness of the insulator-to-metal transition of VO_2_, and the speed of this transition. $\varepsilon_{H}$ values exceeding 0.8 could also be achieved for VO_2_-based SRDs, along with a Δε exceeding that of doped LMO-based ceramic tile SRDs. Finally, the design of the VO_2_-based multilayer structure could be tailored to adapt the emittance tunability at different ranges of wavelengths depending on the intended application (passive thermal control of spacecrafts, furtivity, etc.). Nonetheless, while doped LMO-based ceramic tiles SRDs have proven to be stable under proton, electron and UV irradiation [37], the stability of the very thin VO_2_ layer in VO_2_-based SRDs under a simulated space environment remains relatively unknown, and additional protective layers might be needed to prevent VO_2_ from deterioration. As previously discussed, the top “cold mirror” could also play a role in protecting VO_2_ from degradation in space if designed using space-compatible materials, such as the hydrogenated a-Si deposited by PECVD used in the present study.

## 4. Conclusions

In this work, I have demonstrated that a combined optical simulation/experimental approach can be used to further optimize the characteristics of VO_2_-based SRDs for effective application in passive thermal control of spacecrafts in general, and nanosatellites in particular. More specifically, I have confirmed that through the addition of a top stack alternating high–low refractive indices consisting of a-Si(25)/SiO_2_(67 nm) on VO_2_(20 nm)/SiO_2_(1330 nm)/Au(300 nm) SRD, the solar absorptance was reduced by 35% (from 0.43 to 0.28) while keeping the emittance performance of the SRD within the requirements for the intended application, with $\varepsilon_{L}$ = 0.35, $\varepsilon_{H}$ = 0.81 and Δε = 0.46. I have also discussed factors that should be considered for further experiments aiming toward the improvement in SRD performance and proposed potential solutions to achieve it. For instance, reducing the roughness of the layers and/or avoiding any change in the material properties of the underlying layers when processing additional top layers could be achieved through an appropriate selection of the thin film deposition methods and/or deposition parameters (such as substrate temperature). Additionally, exploring space-compatible, infrared-transparent materials other than SiO_2_ could help in not only keeping the largest Δε possible when adding extra stack layers (i.e., patterns) alternating between high and low refractive indices to reduce the solar absorptance to below 0.2, but also in protecting VO_2_ from degradation in the space environment.

## Data Availability

Not applicable.

## Supplementary Materials

The following supporting information can be downloaded at: https://www.mdpi.com/article/10.3390/nano12244422/s1, Figure S1: SEM Cross-section image of a-Si layer deposited at 350°C using PECVD on a quartz substrate; Figure S2: SEM Cross-section image of SiO_2_ layer deposited at 390°C using PECVD on a silicon wafer; Figure S3: SEM Cross-section image of VO_2_ layer deposited at room temperature using Pulsed Laser Deposition on a silicon wafer.
