# Supplementary material for: Low Solar Absorptance, High Emittance Performance Thermochromic VO2-Based Smart Radiator Device
Source: Nanomaterials (Basel). 2022 Dec 11;12(24):4422. doi: 10.3390/nano12244422 (PMC9783994; doi:10.3390/nano12244422)
Supplement: Supplementary file 1 [file nanomaterials-12-04422-s001.zip › nanomaterials-2021212-supplementary.pdf]

Low Solar Absorptance, High Emittance Performance Thermochromic VO_2-Based Smart Radiator Device

Ali Hendaoui

Physics Department, College of Science and General Studies, Alfaisal University, P.O. Box 50927, Riyadh 11533, Saudi Arabia; ahendaoui@alfaisal.edu

Determination of the deposition rate of a-Si, SiO_2, and VO_2

Amorphous silicon (a-Si):

Plasmalab system 100 PECVD (Plasma Enhanced Chemical Vapor Deposition) from Oxford Instruments was used to deposit a-Si layer using a gas mixture of SiH_4 and Ar using a pre-established recipe in the system for low-rate deposition. The substrate temperature is 350°C. Figure S3 represents the cross-section image of a layer deposited for 20 minutes on a quartz substrate. A thickness of about 540 nm is obtained. This corresponds to a deposition rate of approximately 0.45 nm/s for a-Si.

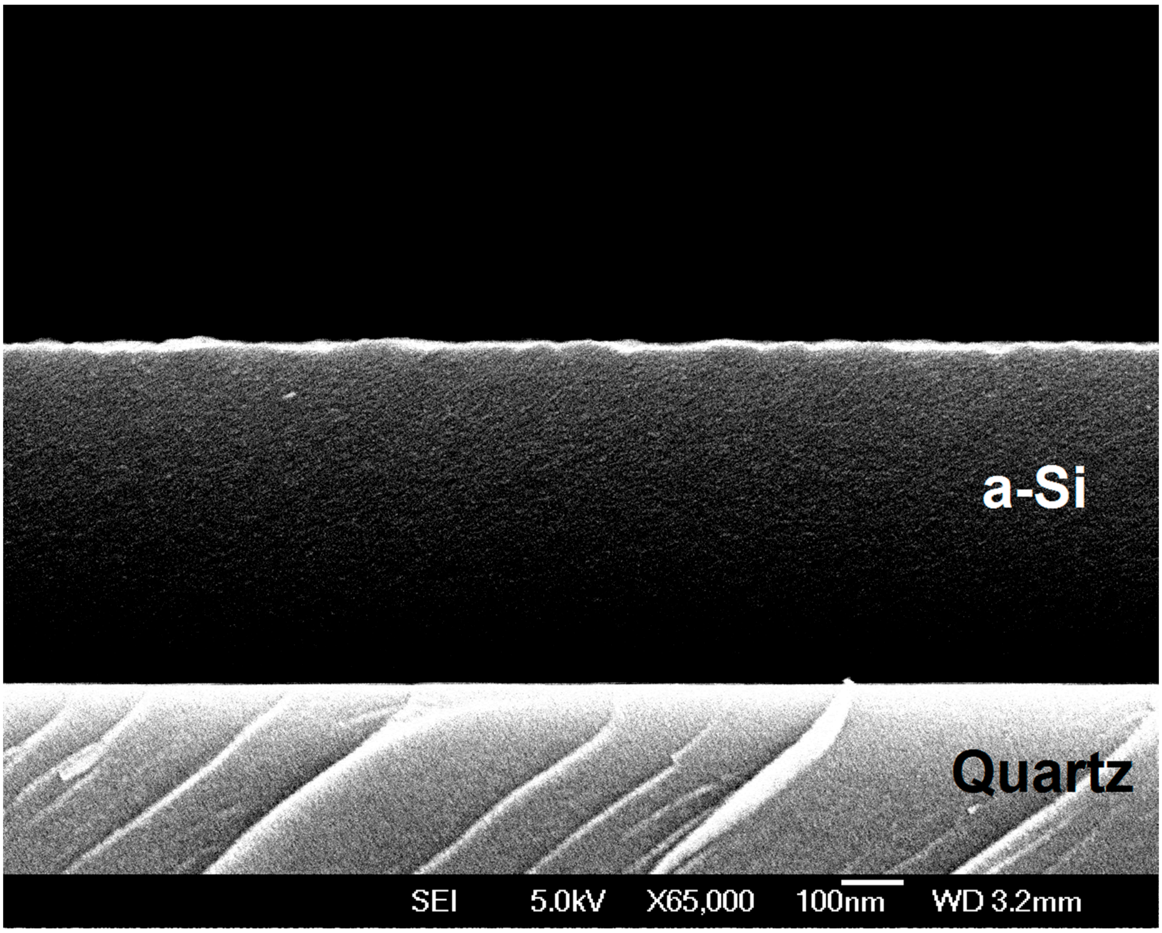

Figure S1. SEM Cross-section image of a-Si layer deposited at 350°C using PECVD on a quartz substrate. The thickness corresponds to a deposition time of 20 minutes.

Silicon dioxide SiO_2:

Plasmalab system 100 PECVD (Plasma Enhanced Chemical Vapor Deposition) from Oxford Instruments was used to deposit SiO_2 layer using a gas mixture of SiH_4, N_2O and N_2 using a pre-established recipe in the system. The substrate temperature is 390°C. Fig S1 represents the cross-section image of a layer deposited for 15 minutes on a silicon wafer substrate. A thickness of about 900 nm is obtained. This corresponds to a deposition rate of approximately 1 nm/s for SiO_2.

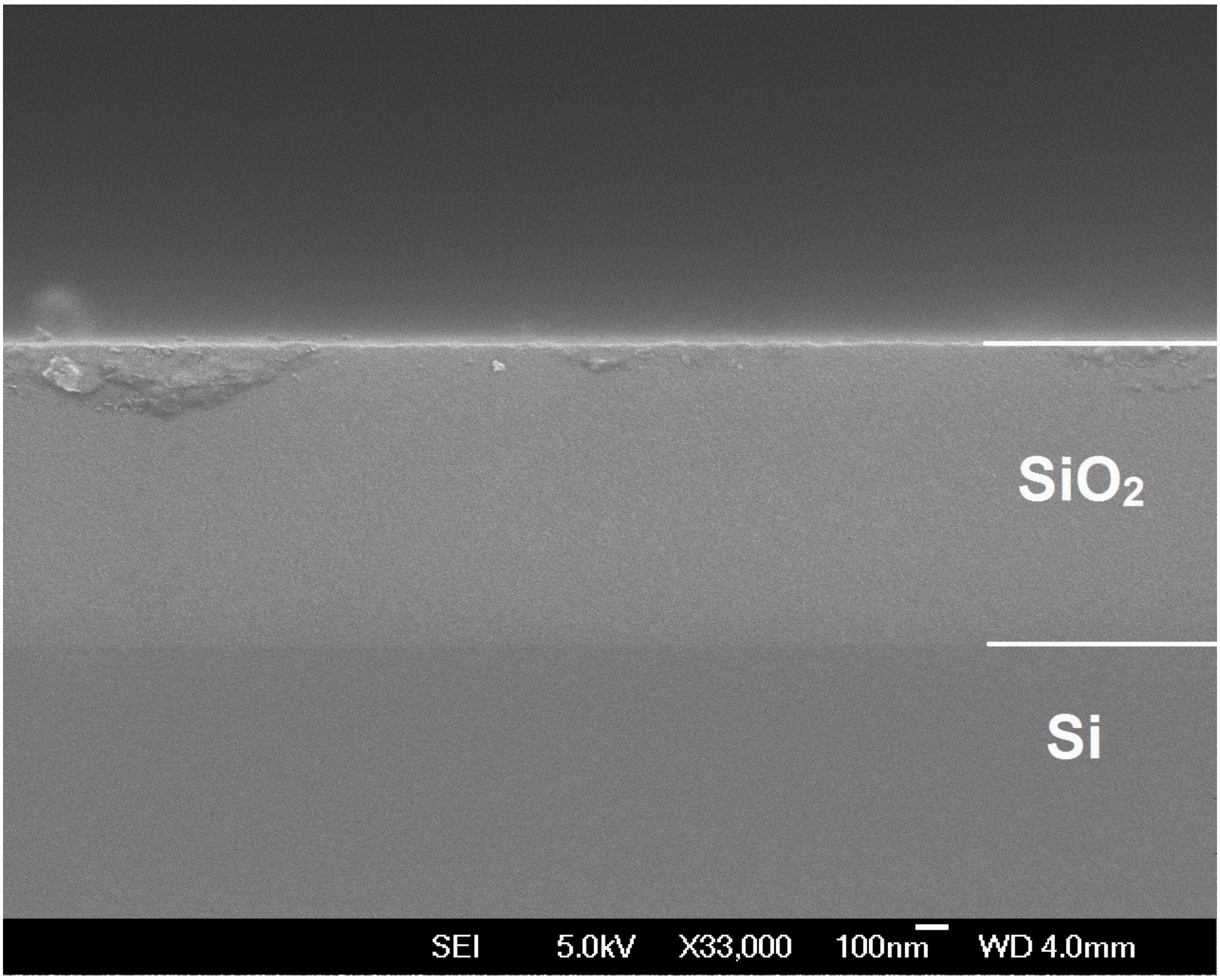

Figure S2. SEM Cross-section image of SiO_2 layer deposited at 390°C using PECVD on a silicon wafer. The thickness corresponds to a deposition time of 15 minutes.

Vanadium dioxide VO_2:

VO_2 film was deposited on a silicon wafer using a KrF excimer pulsed laser (λ = 248 nm, laser fluence equal to 1.8 $J.cm^{-2}$) focused on Vanadium target (99.9% pure) under 5 mTorr of oxygen ambient. The laser was pulsed at a frequency of 2 Hz. To establish the deposition rate accurately, the process was performed at room temperature (substrate temperature at 20 to 25°C). the deposition time was set at 5 hours (i.e., 36000 pulses). Figure S3 represents the cross-section image of the layer. A thickness of about 200 nm is obtained. This corresponds to a deposition rate of approximately 0.01 nm/s for VO_2.

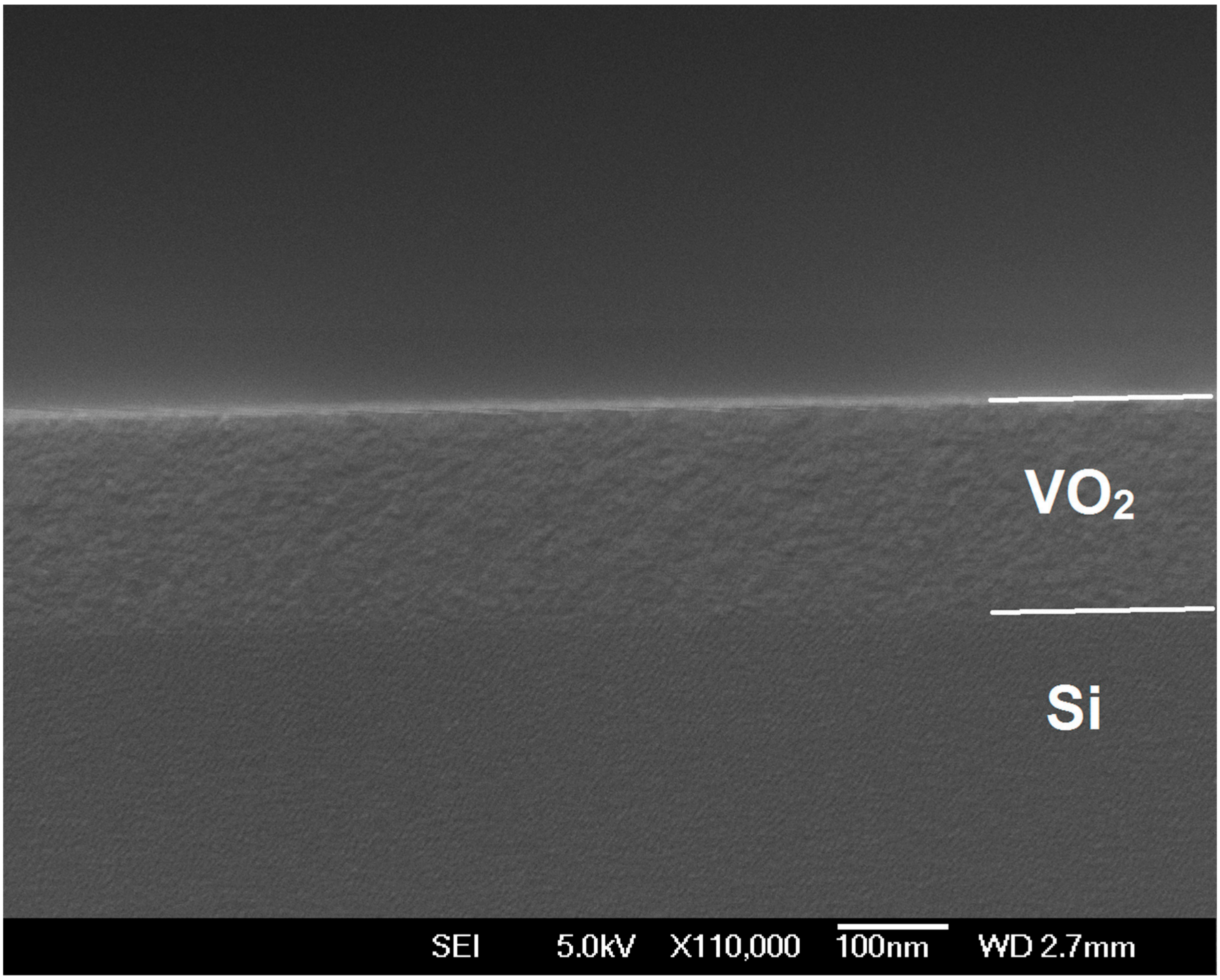

Figure S3. SEM Cross-section image of VO_2 layer deposited at room temperature using Pulsed Laser Deposition on a silicon wafer. The thickness corresponds to a deposition time of 5 hours.
